# Job satisfaction and its associated factors among health care workers in Saint Paul’s Hospital Millennium Medical College, Ethiopia

**DOI:** 10.1371/journal.pone.0326496

**Published:** 2025-06-25

**Authors:** Melese Bahiru Tesema, Berhanu Teshome Woldeamanuel, Amideyesus Adinaw Lopiso, Muluye Abebe Beyene

**Affiliations:** 1 Research Training Quality Control Division, Research Training Directorate, Armauer Hansen Research Institute, Addis Ababa, Ethiopia; 2 Department of Epidemiology and Biostatistics, School of public health, Saint Paul’s Hospital Millennium Medical College, Addis Ababa, Ethiopia; 3 Director, Research Training Directorate, Armauer Hansen Research Institute, Addis Ababa, Ethiopia; 4 Genomics and Bioinformatics, Armauer Hansen Research Institute, Addis Ababa, Ethiopia; SPHMMC: St Paul's Hospital Millennium Medical College, ETHIOPIA

## Abstract

**Background:**

Job satisfaction, a positive emotional state from evaluating work experiences, has a global prevalence of 46.68% among healthcare workers, often lower than other public servants due to challenging conditions. Improving job satisfaction is vital for better healthcare services. However, information on the level of job satisfaction among health professionals in Ethiopia remains scarce. This study sought to assess job satisfaction and its associated factors among health professionals at Saint Paul’s Hospital Millennium Medical College in Addis Ababa, Ethiopia.

**Methods:**

An institution-based cross-sectional study was conducted from April 03/2023 to May 10/2023, involving 439 randomly selected healthcare workers. Data collection utilized the interviewer-administered Minnesota Satisfaction Questionnaire. Ordinal logistic regression was applied to identify factors associated with job satisfaction. Statistical significance was determined by a p-value of less than 0.05 or a 95% confidence interval for the adjusted odds ratio excluding one.

**Results:**

All 439 healthcare workers participated, achieving a 100% response rate. Job satisfaction levels were categorized as very dissatisfied (27.1%), dissatisfied (26.2%), satisfied (24.8%), and very satisfied (21.9%). Factors associated with decreased job satisfaction included a monthly salary below 8,000 ETB (AOR = 0.377, CI: 0.174–0.814), salary of 8,000–10,000 ETB (AOR = 0.249, CI: 0.117–0.571), history of headaches (AOR = 0.607, CI: 0.406–0.908), working in emergency (AOR = 0.205, CI: 0.084–0.502), inpatient (AOR = 0.391, CI: 0.167–0.916), or radiology environments (AOR = 0.081, CI: 0.013–0.489). Increased job satisfaction was linked to nursing (AOR = 1.679, CI: 1.011–2.787) and radiology professions (AOR = 35.21, CI: 4.14–299.39).

**Conclusion:**

Factors such as low salary and high-stress work environments reduce job satisfaction, necessitating strategies to improve workplace conditions and revise salary structures to foster greater satisfaction among healthcare workers. The hospital should address high-stress environments like emergency and inpatient settings and offer support to healthcare workers with headaches. Moreover, the Ministry of Health should revise salary structures and assess work environments to enhance healthcare workers’ job satisfaction.

## Background

Job satisfaction is a pleasant or positive attachment state developed as a person evaluates their work experience [1]. Further, it is described as the employee’s subjective assessment of their working environment or situations [2]. On the other side, job dissatisfaction refers to an employee’s emotional response to their work experience as a result of difficult working conditions or environment [3]. Because dissatisfied employees often lack ambition, perform poorly, and have unfavorable attitudes, they can have a detrimental impact on the quality of health care of the organization [4].

Health care workers (HCWs) job satisfaction levels are closely related to the quality of health services given [5], and due to the nature of their working conditions, there is more job dissatisfaction among HCWs than general public servants [6–8]. The level of job satisfaction varies from 15% to 80.8% in developed nations [9]. A global, meta-analysis and systematic review of studies on job satisfaction among general practitioners show the pooled level of job satisfaction to be 70.82%, and it dropped from an average of 72.39% before 2009 to 63.09% later in 2020 [10]. A similar study done in Chhattisgarh, India, using a job satisfaction survey scale instrument among 400 HCWs shows the level of job satisfaction to be 15% among doctors and 6.67% among nurses, and it further shows that medical doctors were more dissatisfied (20%) than nurses (6.67%) [11]. Further, study done in China shows the extent of job satisfaction to be 74.33% [12].

A study done in Brazil shows that the extent of job dissatisfaction to be 25%. Further, it shows that 66% of HCWs already thought of leaving the profession [13]. Another study done in one of the university hospitals in Vietnam shows 43.1% of HCWs satisfied with their job [14]. Further a cross-sectional study done in the northwest province of South Africa among 244 HCWs shows 38% of respondent reported being satisfied with their job [6]. Furthermore, a study done in Nigeria shows the level of job satisfaction to be 46.1% in tertiary teaching hospitals [15], 70.1% in Zaria tertiary hospitals [16], 88.9% among primary health care workers [17], and 90.4% in Kano tertiary hospital [18]. Moreover, a study done in Ghana and Kenya shows the satisfaction rate to be 38.1% [19].

In Ethiopia, a systematic review and meta-analysis study reported a pooled satisfaction rate of job satisfaction from 35 studies among HCWs was 46.68%. Further, by profession, it was found to be 16.5%, 39.2%, 44.56%, 48.57%, 48.8%, and 57.56% among health extension workers, physicians, anaesthetists, nurses, midwives, health officers, and pharmacy professionals, respectively [7]. Another study done in Bahir Dar and West Shoa, Oromia, Ethiopia; the overall level of job satisfaction was found to be 55.2% [20] and 34.9% [21], respectively. In one of tertiary hospital in Addis Ababa, the job satisfaction was found to be 47.7% [8].

Regarding the risk factors related with job satisfactions among HCWs age, autonomy, adequate supportive supervision, good reward and recognition, year of experience, working environment, work load, lack of professional growth opportunities, relationships with co-workers and supervisors, interpersonal communication, salary, and promotional opportunities and having Depressive symptoms were among the common listed [6], [11], [13], [20], [22–27].

Despite the health sector in Ethiopia shows a significant improvement, health care workers job satisfaction has remained stable low. Different studies had examined job satisfaction among healthcare workers in Ethiopia, but this study focuses on Saint Paul’s Hospital Millennium Medical College, a leading teaching and referral institution in Ethiopia with unique organizational dynamics. Its distinct culture, evolving healthcare demands, and workforce composition call for context-specific evidence to inform effective interventions. This research provides up-to-date insights into key factors such as workload, and workplace environment offering understanding of their impact on job satisfaction. By addressing these elements within a localized setting, the study not only fills knowledge gap but also support to develop strategies to enhance employee well-being, improve patient care quality, and strengthen institutional performance. Therefore, this study aims to assess the level of job satisfaction and its associated factors among health care workers at Saint Paul’s Hospital Millennium Medical College (SPHMMC), Addis Ababa, Ethiopia.

## Methods

### Study design and setting

A facility-based cross-sectional study was conducted SPHMMC in Addis Ababa, Ethiopia, between April and May 2023. The hospital, originally established in 1968 under the reign of Emperor Haile Selassie I and later expanded with a medical school in 2007, was formally restructured and renamed SPHMMC in 2010 through a decree by the Council of Ministers. Operating under the governance of the Federal Ministry of Health’s board, the hospital offers a range of services including preventive, curative, and rehabilitative care to patients referred from health centers. With a capacity of approximately 700 inpatient beds, the hospital serves an average of 1,200 emergency and outpatient cases daily [28].

### Sample size and study population

**Fig 1:**
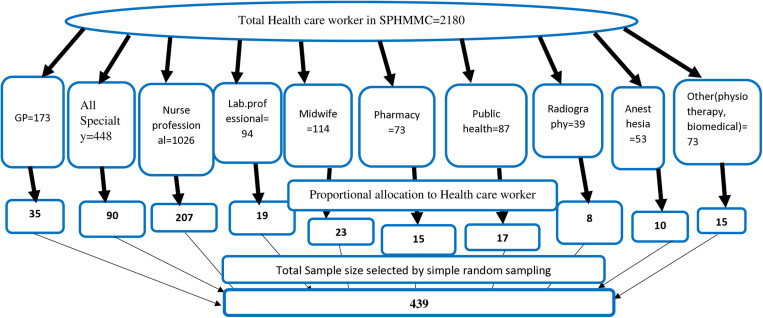
Proportional allocation to study the level of job satisfaction and its associated factors Among Health Care Professionals Working at SPHMMC, Addis Ababa, Ethiopia).

The sample size was determined using Epi Info’s statistical calculator and the double population proportion formula, applying a 95% confidence interval and 80% power. Factors such as odds ratio, the ratio of unexposed to exposed participants, and the proportion of the relevant exposure variable were incorporated, alongside a 10% adjustment for non-response. Specifically, for sex (female), with an odds ratio (OR) of 1.94 and a prevalence of 24.5%, the required sample size was 435 after adjusting for non-response. For marital status (single), an OR of 2.16 and a prevalence of 22.1% led to a required sample size of 326, adjusted to non-response rate. Similarly, a family history of mental illness, associated with a high OR of 7.31 and a prevalence of 24.4%, necessitated a sample size of 161 after accounting for non-response. Smoking status, characterized by an OR of 2.67 and a prevalence of 22.2%, required a sample size of 244 after adjustment. Finally, workload (hours), with an OR of 1.997 and a prevalence of 69%, necessitated a sample size of 439 after including a 10% allowance for non-response, which represented the largest sample size and was therefore selected as the final sample for this study. Given the internal homogeneity and external heterogeneity of healthcare workers across professions, stratified random sampling was employed. Professions served as the basis for stratification, and simple random sampling within each stratum ensured proportional allocation to each stratum’s size. To undertake simple random sampling technique, we first gave each participant a unique number in each stratum. Next, we used a random number generator to choose the designated sample numbers throughout each stratum at random, and the participants who matched these numbers were included in our study’s sample. If the matched participant is not presented we replaced the absent individual with another randomly selected person from the remaining pool who was not originally chosen (Fig 1).

## Measurement of study variables

### Dependent variable

Job satisfaction, serving as the outcome variable for this study, was assessed through the application of the short-form Minnesota Satisfaction Questionnaire (MSQ), specifically tailored to evaluate HCWs [29], [30]. The reliability test in the previous study revealed that the tool for the subscale was reliable, with a Cronbach’s alpha score of 0.87 [30]. In this study Cronbach’s alpha score was found to be 0.892. However, direct validation studies in Ethiopia are limited. The short form of the MSQ comprises 20 items, each designed to yield a cumulative score ranging from 20 to 100. Each item within the scale was weighted on a Likert scale ranging from 1 to 5, with responses scored sequentially from left to right in the answer space. These scores corresponded to the following levels of satisfaction: 1-Very Dissatisfied, 2-Dissatisfied, 3-Neutral, 4-Satisfied, and 5-Very Satisfied. The total row score for each scale was calculated by summing the scores assigned to all five response categories within the scale, which subsequently facilitated the determination of percentile scores. Satisfaction levels were categorized based on these percentile scores: a score of 75 or higher denoted a higher degree of satisfaction, a score ranging from 50 to 74 indicated satisfaction, a score between 26 and 49 reflected dissatisfactions, and a score below 25 represented a very dissatisfied [29]. This systematic approach ensured the accurate quantification and classification of job satisfaction among HCWs, enabling meaningful analysis and interpretation of the data [29].

### Independent variables

The exposure variables were sociodemographic variables (age, sex, marital status, religion, level of education and monthly income), and psycho-social factors (history of childhood abuse, stressful life event, history of suicidal attempt, presence of family history of mental illness). Further, physical health status (history of injury, presence of disability, headache over past 30 days, back pain over the past 30 days, fever over past 30 days, medical problem, smoking history and social support), health professional status (field of study, working hour, work experience, work environment, working department) were used. The Patient Health Questionnaire (PHQ-9) was used to assess depressive symptoms with nine items of major depressive disorder symptoms from diagnostic and statistical manual-IV criteria. Each item response was rated as 0 = “not at all” to 3 = “nearly every day”. Total score of nine items of PHQ-9 range from 0 to 27. Scores were defined by 0−4 no depression, 5−9 mild, 10−14 moderate, and 15 or above were severe depression, respectively [31]. PHQ-9 was validated previously with sensitivity of 86% and specificity of 67% in Ethiopian population [32]. For the purpose of this study, the cut point of 5 or above was used as depression and below 5 no depression [31].

To assess social support Oslo-3-item social support scale was used and those with score of 3−8 was classified as poor social support, 9−11 as moderate social support and 12−14 as strong social support [33]. Studies had shown good predictive validity regarding psychological problems [33,34]. Further, in African context the scale had acceptable validity and reliability [35]. However, specific validation studies conducted in Ethiopia are not readily available. One study in Ethiopia did use the Oslo-3-item scale alongside other validated tools to assess sleep quality among pregnant women, which suggests some level of applicability in Ethiopian contexts [36]. Limitations may include cultural biases, differences in interpretation, and lack of standardized norms for the Ethiopian population.

The study utilizes Ethiopia’s national poverty line, alongside international poverty thresholds, as a foundational benchmark, noting that the World Bank frequently designates $2.15 per day, adjusted for purchasing power parity, as the extreme poverty line [37,38] a comparison against which the monthly income of 8000 Birr serves to establish a compelling rationale for income classification. Furthermore, the categorization of countries by the World Bank into income groups based on Gross National Income per capita although a more encompassing measure provides a broader contextual framework within which Ethiopia’s income levels may be analyzed [39]. Simultaneously, taking into account the cost of living in Addis Ababa, including essential expenses such as housing, food, and transportation, it becomes evident that if 8000 Birr sufficiently meets or exceeds the basic needs of an average household, it can reasonably be classified as an appropriate income threshold. Additionally, Ethiopia’s Multidimensional Poverty Index, which incorporates factors such as health, education, and living standards, underscores the necessity of aligning income classifications with these dimensions to substantiate their relevance to job satisfaction [40]. Therefore, these considerations collectively provide a robust justification for the income classification utilized within the study.

### Data Collection

Two trained health professionals were enlisted for data collection under the supervision of two authors. A pre-test, encompassing 5% of the health professionals, was carried out at Ras Desta Damtew Referral Hospital, located near SPHMMC. The questionnaire was administered through interviews, and measures were taken to ensure the data’s completeness and consistency, thereby minimizing systematic errors.

### Statistical Analysis

Microsoft Excel version 19 was utilized for data cleaning and verification prior to exporting the dataset to statistical package for social science version 26 for coding and analysis. The findings were summarized descriptively. A bivariable and multivariable ordinal logistic regression model was employed to assess the relationship between dependent and independent variables, with only those factors showing significant association in the bivariable analysis (p-value < 0.25) being included in the multivariable analysis. Adjusted odds ratios with 95% confidence intervals and p-values below 0.05 were considered statistically significant.

Given the ordinal nature of job satisfaction levels, ordinal logistic regression was employed, which operates under the assumption that the relationship between independent variables and the dependent variable remains consistent across all ordinal thresholds. Specifically, the odds ratio for transitioning between consecutive levels of satisfaction for instance, from “very dissatisfied” to “dissatisfied”, “dissatisfied” to “satisfied,” and “satisfied” to “very satisfied” is presumed to be comparable. This assumption was rigorously tested using a chi-square test (Chi-square = 88, p-value = 0.015) and confirmed with no violations detected. Furthermore, multicollinearity among the independent variables was assessed using the Variance Inflation Factor (VIF), with results indicating the absence of multicollinearity issues, as all VIF values were below the critical threshold of 10.

### Ethical consideration

The study protocol was reviewed and approved by the ethical review board of SPHMMC, with ethical clearance granted under Reference No: Pm23/651. Authorization to approach healthcare providers was obtained through a clearance letter from the head of SPHMMC’s research directorate. Measures were taken to ensure confidentiality, and participants’ rights to withdraw or refuse participation were fully respected. Verbal informed consent was secured from participants prior to their involvement in the study. Further, the integrity of the verbal informed consent process was maintained through observing individual engagement such as asking questions and their voluntariness in deciding to participate freely and then at the end the date and time of data collection was documented.

## Result

### Descriptive Statistics

Of the HCWs who took part in the study, 241 (55%) were men, making up more than half. The HCWs were 31.23 years old on average, with a standard deviation of 6.23. Of those surveyed, 220 (50.11%) were unmarried, 206 (46.9%) were married, and 9 (2.1%) were divorced. In terms of educational attainment, 13 (3%), 236 (53.8%), 63 (14.4%), and 127 (28.9%) had college degrees, master’s degrees, and doctorates degrees, respectively. In addition, 49 (11.2%) were Muslims, 93 (21.2%) were Protestants, and 292 (66.5%) were Orthodox. Additionally, with regard to monthly salary, 202 (46%) of the HCWs received less than 8,000 Ethiopian Birr (ETB), or 141.72 United states dollar (Table 1).

**Table 1:** Distribution of socio-demographic variables among HCWs at SPHMMC, Addis Ababa, Ethiopia, 2023(n = 439).

Variable	Category	%(n)
Sex	Male	54.9 (241)
	Female	45.1 (198)
Age	18-25 year	10 (44)
	26-30 year	49.4 (217)
	31-35 year	20.3 (89)
	36-40 year	13 (57)
	41-60 year	7.3 (32)
Marital status	Single	50.1(220)
	Married	46.9(206)
	Divorced	3 13)
Level of education	College Diploma	3 (13)
	Bachler Degree	53.8(236)
	Master Degree	14.4(63)
	Doctorate Degree	28.9(127)
Religion	Orthodox	66.5(292)
	Protestant	21.2(93)
	Muslim	11.2 (49)
	Other	1.1 (5)
Monthly salary	<8,000ETB	46(202)
	8,001–10,000 ETB	25.7(113)
	10,001–14,999ETB	18.9(83)
	>15,000ETB	9.3 (41)

In terms of psychosocial factors, twenty percent of the study participants experienced abuse as children, 51 (11.6%) had a family history of mental illness, 36(8.2%) had attempted suicide, and 212 (48.3%) had experienced stressful life events. Additionally, among those who experienced abuse as children, 34 (38.2%) experienced physical abuse, 26 (29.2%) verbal abuse, and 22 (24.7%) emotional abuse. Furthermore, among individuals who experienced stressful life events 17 (30.9%) were brought on by the loss of money or materials, and 25 (45.5%) were brought on by the death of a close relative or loved one (Table 2).

**Table 2:** Distribution of psychosocial factor among HCWs at SPHMMC,2023 (n = 439).

Variable	Category	% (n)
History of childhood abuse	Yes	20.5(90)
	No	79.5(349)
Presence of family history of mental illness	Yes	11.6 (51)
	No	88.4(388)
History of suicidal attempt	Yes	8.2 (36)
	No	91.8(403)
History of stressful life event	Yes	48.3(212)
	No	51.7(227)

Regarding the physical status and social support, 45(10.3%) had sustained injury, 11(2.5%) had a disability, and 42(9.6%) had a medical problem. Additionally, 146(33.3%) of the study HCWs had headaches, 151(34.4%) had back pain, and 47(10.7%) had fever over the past 30 days, respectively. Further, 31(7.1%) of the study HCWs had smoking history. According to the Oslo 3-item social support scale, those who have poor, moderate, and strong social support constitute 156(35.5%), 223(50.8%) and 60(13.7%) of the study HCWs, respectively (Table 3).

**Table 3:** Distribution of physical health status and social support among HCWs at SPHMMC, 2023(n = 439).

Variable	Category	%(n)
History of Injury	Yes	10.3 (45)
	No	89.7(394)
Presence of disability	Yes	2.5 (11)
	No	97.5 (428)
Presence of head ache over the past 30 days	Yes	33.3(146)
	No	66.7(293)
Presence of back pain over the past 30 days	Yes	34.4(151)
	No	65.6(288)
Presence of fever over the past 30 days	Yes	10.7 (47)
	No	89.3(392)
Presence of medical problem	Yes	9.6 (42)
	No	90.4(397)
Smoking history	Yes	7.1 (31)
	No	92.9(408)
Social support	Poor	35.5(156)
	Moderate	50.8(223)
	Strong	13.7(60)

Nearly half of the study participants were Nurses. There were 35 (8%), general practitioners, and 90 (20.5%), medical specialties, including residency. Regarding other occupations, there were 23 (5.2%) midwives, 19 (4.3%) medical labs, 17 (3.9%) public health, 15 (3.4%) pharmacist, 10 (2.3%) anesthesiologists, 8 (1.8%) radiologists, and 15 (3.4%) others (including psychiatry professionals, phlebotomists, biomedical engineers, and biomedical technicians).

In relation to the years of professional experience of the study participants, 146 (33.3%) had 10–15 years of experience, and 191 (43.5%) had 2–5 years. Regarding the working department and environment, 88 (20%) work in the medical department, 79 (18.3%) work in the surgical department, 13 (3%) work in the ophthalmic department, and 11 (2.5%) work in the ear, nose, and throat department. Additionally, 190 (43.3%) work in the inpatients environment, while 95 (21.6%) work in emergency environment. In addition, 278 (63.3%) of respondents work more than eight hours a day, and 248 (56.4%) reported having some kind of depressive symptoms (Table 4).

**Table 4:** Distribution of professional factor, depression and work area factor among HCWs at SPHMMC,2023(n = 439).

Variable	Category	%(n)
Field of study	Nursing	47.2(207)
	Midwifery	5.2 (23)
	Medical doctor	8 (35)
	Specialty	20.5(90)
	Medical laboratory	4.3 (19)
	Pharmacy	3.4 (15)
	Anesthesia	2.3 (10)
	Public health	3.9 (17)
	Other	3.4 (15)
	Radiology	1.8 (8)
Working hour	More than 8 hours	63.3(278)
	8 hours	31.4(138)
	Less than 8 hours	5.2 (23)
Work experience	Less than 2 years	7.5 (33)
	2-5 year	43.5(191)
	5-10 year	33.3(146)
	10-15 year	10.9 (48)
	Above 15 years	4.8 (21)
Working department	Surgical	18(79)
	Medical	20(88)
	Pediatrics	15.3(67)
	Obstetrics	16.9(74)
	Trauma and burn center	7.1 (31)
	Laboratory	3.9 (17)
	Pharmacy	3.4 (15)
	Ophthalmology	3 (13)
	ENT	2.5 (11)
	Radiology	3.4 (15)
	Psychiatry	2.1 (9)
	Other	2.1 (9)
	Anesthesia	2.5 (11)
Work Environment	Emergency	21.6(95)
	Outpatient	24.6(108)
	Inpatient	43.3(190)
	Radiology	3.4 (15)
	Office	7.1 (31)
Depressive symptoms	Depressive symptoms	56.4(248)
	No depressive symptoms	43.6(191)

### Prevalence of Job Satisfaction among Health Care Worker at SPHMMC

**Fig 2:**
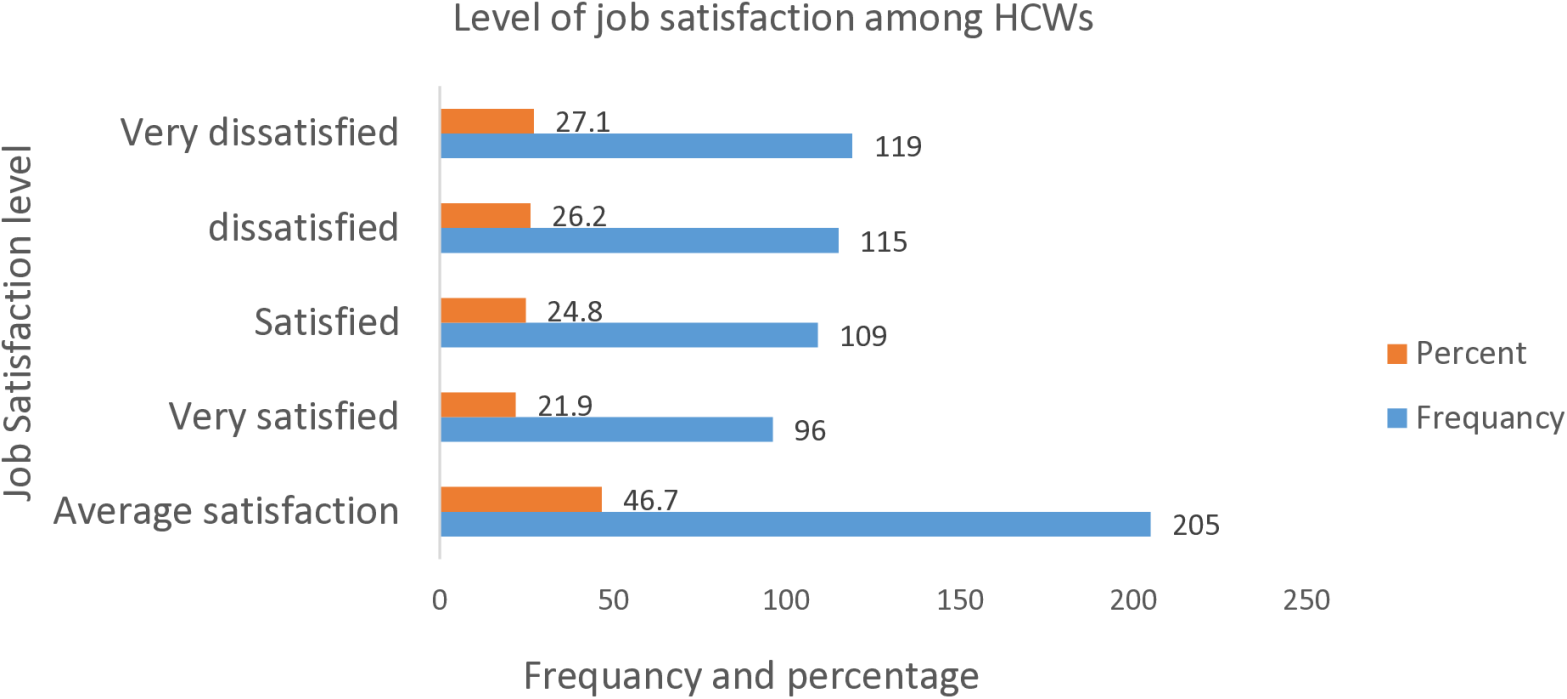
Level of job satisfaction among HCWs, in SPHMMC, Addis Ababa, Ethiopia.

Based on the MSQ scale, the overall prevalence of job satisfaction among HCWs at SPHMMC was found to be 46.7% (95% CI: 42%, 51.3%). Of which 117 (57%) were male. Concerning the level of satisfaction, 119 (27.1%) were very dissatisfied, 115(26.2%) were dissatisfied, and 109(24.8%) were satisfied and 96(21.9%) were very satisfied. The overall level of job satisfaction among HCWs at the SPHMMC was low (Fig 2).

### Factors associated with job satisfaction among Health Care Worker at SPHMMC

The final model was obtained by first fitting a bivariable logistic regression, and then using backward elimination techniques to incorporate all candidate predictors significant at 25% in the bivariable analysis for the multivariable analysis. The LRT of goodness-of-fit test (Chi-square = 87.46) with p-value <0.001 was used to evaluate the model’s overall goodness-of-fit test which indicated that the model was well-fitting. After fitting the proportional odds model, the LRT was used to verify that the proportionality assumption was met. The results showed no violations (Chi-square = 88, p-value = 0.015). Using VIF, multi-collinearity was examined. No multicollinearity issue was discovered.

The odds of job satisfaction were shown to be significantly decreased for monthly salaries below 8,000 ETB (AOR = 0.377, 95% CI (0.174, 0.814)) and between 8000 and 10,000 ETB (AOR = 0.249, 95% CI (0.117, 0.571)) than for monthly salaries over 15,000 ETB. Additionally, compared to individuals without a history of headaches, those who had a history of headaches one month earlier had a decreased odds of job satisfaction (AOR = 0.607, 95% CI (0.406, 0.908)). Additionally, compared to those who work in offices, HCWs who work in emergency work environment (AOR = 0.205, 95% CI (0.084, 0.502)), inpatient (AOR = 0.391, 95% CI (0.167, 0.916)), and radiology work environment (AOR = 0.081, 95% CI (0.013, 0.489)) had decreased odds of job satisfaction. In contrast, HCWs who pursued careers in nursing (AOR = 1.679, 95% CI (1.011, 2.787)) were more likely to be in greater odds of job satisfaction than those who pursued careers in pharmacy, laboratory, or midwifery. Similarly, HCWs who worked as radiologists (AOR = 35.21, 95% CI (4.14,299.39)) were more likely to be in greater odds of job satisfaction than those who pursued careers in pharmacy, laboratory, or midwifery (Table 5).

**Table 5:** Multivariable analysis of different variable and job satisfaction among HCWs of SPHMMC Addis Ababa, Ethiopia,2023(n = 439).

Variable	Category	Job Satisfaction level				P-Value	COR (95%CI)	P-Value	AOR (95%CI)
		Very dissatisfied % (n)	Dissatisfied %(n)	Satisfied %(n)	Very Satisfied %(n)				
Age	18-25 year	30 (13)	36 (16)	25 (11)	9 (4)	0.004	0.307(0.132,0.687)	0.888	1.095(0.308,3.899)
	26-30 year	30(66)	29(63)	22 (48)	18 (40)	0.004	0.366(0.184,0.731)	0.893	1.073(0.382,3.014)
	31-35 year	24 (21)	25 (22)	30 (27)	55 (49)	0.082	0.517(0.246,1.087)	0.699	0.825(0.311,2.186)
	36-40 year	25 (14)	12 (7)	28 (16)	35 (20)	0.586	0.799(0.357,1.790)	0.846	1.103(0.409,2.971)
	41-60 year	16 (5)	22 (7)	22 (7)	41 (13)		1.00		1.00
Marital status	Single	33(72)	26(57)	25 (55)	16 (36)	0.091	0.34(0.097,1.189)	0.966	1.03(0.261,4.07)
	Married	22 (46)	27 (55)	24 (49)	27 (56)	0.367	0.56(0.161,1.965)	0.631	1.385(0.366,5.243)
	Divorced	8 (1)	23 (3)	38 (5)	31 (4)		1.00		1.00
Monthly salary in ETB	<8000	31(63)	24 (49)	25 (51)	19 (39)	0.001	0.366(0.197,0.679)	0.01**	**0.377(0.174,0.814)**
	8,001-10,000	28 (32)	30 (34)	27 (31)	14 (16)	0.001	0.343(0.178,0.659)	0.001**	**0.249(0.117,0.517)**
	10,001-14,999	23 (19)	27 (22)	22 (18)	29 (24)	0.082	0.543(0.273,1.080)	0.062	0.502(0.244,1.036)
	>15,000	12 (5)	24 (10)	22 (9)	41 (17)		1.00		1.00
History of suicidal attempt	Yes	42 (15)	11 (4)	31 (11)	17 (6)	0.250	0.687(0.363,1.302)	0.221	0.648(0.324,1.298)
	No	26(104)	28(111)	24(98)	22(90)		1.00		1.00
History of stressful life event	Yes	29(61)	26 (56)	26 (55)	19 (40)	0.239	0.817(0.585,1.143)	0.682	1.081(0.745,1.569)
	No	26(58)	26(59)	24 (54)	25 (56)		1.00		1.00
Presence of headache over the past 30 days	Yes	37 (54)	25 (37)	21 (30)	17 (25)	0.001	0.551(0.383,0.792)	0.015*	**0.607(0.406,0.908)**
	No	22(65)	27(78)	27(79)	24(71)		1.00		1.00
Field of study	Nursing	25 (51)	25 (52)	30(62)	20 (42)	0.063	1.491(0.978,2.272)	0.045*	**1.679(1.011,2.787)**
	Medical doctor	31 (39)	22 (27)	18 (22)	30 (37)	0.116	1.468(0.910,2.369)	0.663	1.142(0.629,2.077)
	Radiology	0	13 (1)	50 (4)	38 (3)	0.014	4.658(1.361,15.938)	0.001**	**35.21(4.14,299.39)**
	Other	29 (29)	35 (35)	21 (21)	14 (14)		1.00		1.00
Working hour	> 8 hours	30(84)	30(75)	24(66)	19 (53)	0.05	2.158(0.996,4.677)	0.346	0.661(0.279,1.563)
	8 hours	22 (31)	25 (35)	27 (37)	25 (35)	0.041	1.464(1.015,2.111)	0.541	0.760(0.315,1.831)
	< 8 hours	17 (4)	22 (5)	26 (6)	35 (8)		1.00		1.00
Work experience	< 2 years	42 (14)	27 (9)	18 (6)	12 (4)	0.018	0.280(0.097,0.807)	0.776	0.81(0.189,3.46)
	2-5 year	304(58)	28 (53)	25 (48)	17 (32)	0.085	0.458(0.188,1.114)	0.717	1.25(0.374,4.173)
	5-10 year	23 (33)	27 (39)	28 (41)	23 (33)	0.361	0.658(0.267,1.614)	0.506	1.48(0.468,4.669)
	10-15 year	17 (8)	23 (11)	23 (11)	38 (18)	0.838	1.109(0.410,3.003)	0.191	2.188(0.677,7.07)
	Above 15 years	29 (6)	14 (3)	14 (3)	43 (9)		1.00		1.00
Working department	Major	27(91)	26(88)	25(84)	22(76)	0.224	1.924(0.670,5.528)	0.263	1.969(0.601,6.446)
	Minor	27 (24)	26 (23)	26 (23)	21 (19)	0.258	1.889(0.628,5.689)	0.480	1.569(0.450,5.473)
	Anesthesiology	36 (4)	36 (4)	18 (2)	9 (1)		1.00		1.00
Work Environment	Emergency	43 (41)	27 (26)	19 (18)	11 (10)	0.001	0.255(0.116,0.560)	**0.001** ^**^	**0.205(0.084,0.502)**
	Outpatient	21 (23)	21 (24)	29 (31)	28 (30)	0.516	0.775(0.360,1.671)	0.252	0.614(0.267,1.414)
	Inpatient	23 (43)	31(58)	26 (50)	21 (39)	0.138	0.574(0.275,1.196)	**0.031** ^*^	**0.391(0.167,0.916)**
	Radiology	27 (4)	14 (2)	33 (5)	27 (4)	0.618	0.746(0.235,2.364)	**0.006** ^**^	**0.081(0.013,0.489)**
	Office	26 (8)	16 (5)	16 (5)	42 (13)		1.00		1.00
Depression symptoms	Depression	31(78)	27(67)	26(64)	16 (39)	0.001	0.558(0.396,0.785)	0.196	0.77(0.531, 1.138)
	No depression	21 (41)	25 (48)	24 (45)	30(57)		1.00		
Social support	Poor	13 (51)	15 (42)	38 (40)	34 (23)	0.126	0.661(0.389,1.124)	0.573	0.849(0.48,1.49)
	Moderate	8 (54)	13 (56)	32 (54)	47(59)	0.772	1.078(0.649,1.792)	0.282	1.345(0.783,2.311)
	Strong	5 (14)	3 (17)	37 (15)	55 (14)		1.00		1.00

NB:

* =significant at 0.05,

** =significant at 0.01 COR=Crude odd ratio AOR=Adjusted odd ratio CI= Confidence interval n=frequency

## Discussion

This study assessed job satisfaction and its associated factors among HCWs. It was determined that 46.7% (95%CI: 42%, 51.3%) of people were generally satisfied with their jobs. Regarding the level of job satisfaction, 119 (27.1%) were very dissatisfied, 115(26.2%) were dissatisfied, and 109(24.8%) were satisfied and 96(21.9%) were very satisfied. This is nearly agreed with research conducted in Ethiopia 46.17% [41], and 46.68% [7], and Nigeria 46.1% [15].

But, the proportion of job satisfaction, in our study was higher when compared to study done in Brazil 25% [13], Ethiopia 34.9% [21], South Africa 38%[6], Ghana and Kenya 38.1% [19],Vietnam 43.1% [14], and India (15% among doctors and 6.67% among nurses) [11]. Different study designs and tools may be the cause of this disparity; for instance, our study employed the MSQ, while an Indian study used the Job Satisfaction Survey Scale. Another explanation for the disparity could be a difference in sample size. Our study included 439 HCWs, while a study conducted in South Africa had 244 HCWs. The differences in the socioeconomic and demographic characteristics of the members within the communities may also be a contributing factor.

On the other hand, the findings from this study was lower compared to studies done in China 74.33% [12] and Nigeria 90.4% [18]. The inclusion criteria, sample size, and instrument employed may be the cause of the disparity. Furthermore, the discrepancy may be caused by differences in the study participants, time, and working environment, as well as likely differences in a medical setting.

In this study, the following factors were associated to job satisfaction: low salary, history of headaches, work environment (including emergency, inpatient, and radiology), and field of study (nursing and radiology). According to this study, HCWs with monthly salaries under 8,000 ETB and between 8000 and 10,000 ETB had 0.377 and 0.249 times lower odds of being satisfied with their jobs, respectively, than those with salaries over 15,000 ETB. This result was in line with previous results from Jima Ethiopia [42], World Bank [43], Gonder Ethiopia [44], Brazil [24] and Germany [45]. This is due to the fact that when HCWs fairly paid; they can meet their personal and family needs, reduce stress about finance and become committed to their assigned job, give expected quality care to patient and more likely to become loyal and then become satisfied with their job. In the reverse if they can’t accommodate their daily cost and can’t meet their personal and family needs, stress about finance increased. This creates opportunity to give low quality of care to patient, didn’t become committed to assigned job and in turn become dissatisfied on their jobs.

According to this study, those who had a headache history within the previous month reported 0.607 times lower levels of job satisfaction than those who had no headache history. Similarly, a study conducted in Rome, Italy, found that headaches were linked to decreased levels of satisfaction [46]. This is because of those HCWs who suffered from headache feels negative perception toward their headache which leads to burnout and then become dissatisfied on their job.

Further, the work environment had a negative impact on HCWs’ job satisfaction. The odds of job satisfaction among HCWs who worked in emergency environment were 0.205 times lower than that of HCWs who worked in office. Similarly, those who worked in an inpatient setting reported 0.391 times lower levels of job satisfaction than those who worked in an office setting. Additionally, those who worked in radiology work environment had a 0.081times lower level of job satisfaction than those who worked in offices. This is line with other similar studies [10, 11, 20, 34, 36]. This is due to the fact that working at emergency and inpatient set up needs caring of critically ill patient on continued base which in turn increased work load and sense of being stressed which results in decreased job satisfaction. Further working at radiology work environment create sense of low health safety related to radiation exposure which may results in decreased job satisfaction.

Furthermore, this study found that those who were nurse as their field of study were 1.68 times at greater level of job satisfaction than those who study other field of study like pharmacy, laboratory, midwifery and public health. In a similar vein, HCWs who study radiology were 35.2 times at increased level of job satisfaction than those who study other field of study like pharmacy, laboratory, midwifery and public health. This is also stated by other studies in which professional status had effect on job satisfaction [7, 37]. This is because of being either of radiologist or radiographer as field of study involve in diagnosing case of patient which results in increased job satisfaction. Similarly nursing field of study involve caring of patient either in critically or in non-critical ill state as a result they become satisfied when patient in care were improved well.

### Limitation of the study

The cross-sectional design we used prevented the establishment of causal relationships. Since it was conducted in a single institution, the findings may not be generalizable to healthcare workers in other settings. The use of yes-or-no responses for instance to assess chronic illness oversimplified these complex issues, lacking detail on severity and context. Additionally, the use of quantitative methods in this study limited the depth of insight into job satisfaction. A mixed-methods approach could have provided a more comprehensive understanding by incorporating personal experiences and contextual factors.

## Conclusion and recommendations

In this study, we examined the prevalence of job satisfaction and its associated factors among SPHMMC’s HCWs. The results show that HCWs are less satisfied with their jobs. Job satisfaction was significantly associated with HCWs who had a history of headaches, had monthly salary of less than 10,000 ETB, worked in emergency, inpatient, and radiology environment, and had studied nursing and radiology. Thus, the hospital should regularly assess work conditions in emergency, radiology, and inpatient environment to identify stress factors, prioritizing interventions like workload redistribution, improved staffing, and adequate resources, while developing wellness programs to address physical and mental health needs, including tailored support for staff with headache histories through flexible scheduling and medical care. At the Ministry of Health level, compensation structures should be re-evaluated to align with the cost of living and the critical contributions of healthcare workers, alongside initiating a nationwide study on work environments to inform strategic plans for workplace improvements, particularly in high-stress areas. Long-term policies should ensure equitable resource distribution, establish minimum standards for staffing and workload management, and promote healthcare worker well-being.

## Supporting information

S1 File Data. (SAV)

## Abbreviations

AOR: Adjusted odd ratio
COR: Crude odd ratio
CI: Confidence interval
ETB: Ethiopian birr
HCWs: Health care workers
LRT: Likelihood ratio test
PHQ-9: Patient health questionnaire-9
SPHMMC: Saint Paul’s hospital millennium medical college
VIF: Variance inflation factor.
